# RGS2 and female common diseases: a guard of women’s health

**DOI:** 10.1186/s12967-023-04462-3

**Published:** 2023-08-30

**Authors:** Qiang Xu, Mukun Yao, Chao Tang

**Affiliations:** 1 National Clinical Research Center for Child Health of the Children’s Hospital, Zhejiang University School of Medicine, No. 3333, Binsheng Rd, Hangzhou, 310052 People’s Republic of China; 2 Department of Gynecology, Tongde Hospital of Zhejiang Province, Hangzhou, 310012 China

**Keywords:** RGS2, Pregnancy, Cancer, Disease, Female Health

## Abstract

Currently, women around the world are still suffering from various female common diseases with the high incidence, such as ovarian cancer, uterine fibroids and preeclampsia (PE), and some diseases are even with the high mortality rate. As a negative feedback regulator in G Protein-Coupled Receptor signaling (GPCR), the Regulator of G-protein Signaling (RGS) protein family participates in regulating kinds of cell biological functions by destabilizing the enzyme–substrate complex through the transformation of hydrolysis of G Guanosine Triphosphate (GTP). Recent work has indicated that, the Regulator of G-protein Signaling 2 (RGS2), a member belonging to the RGS protein family, is closely associated with the occurrence and development of certain female diseases, providing with the evidence that RGS2 functions in sustaining women’s health. In this review paper, we summarize the current knowledge of RGS2 in female common diseases, and also tap and discuss its therapeutic potential by targeting multiple mechanisms.

## Background

The Regulator of G Protein Signaling (RGS) protein family is a feedback regulator in GPCR signaling, which functions as a common GTPase activating protein (GAP) for G proteins and usually acts to add the natural guanosine triphosphatase (GTPase) activity of the Gα-subunit. Generally, through the enhancement of the GTPase activity of the Gα-subunit, RGS proteins encourage GTP hydrolysis to guanosine diphosphate (GDP), therefore switching the Gα subunit to its devitalized state and decreasing its signaling ability [1]. The family of RGS protein can be roughly classified into two sub-groups: (1) one group is composed mainly of a classical RGS domain with small extensions at both the amino-terminal (N-terminal) and carboxyl-terminal (C-terminal); (2) and one group that possesses several other domains in addition to the RGS domain.

**Fig. 1:**
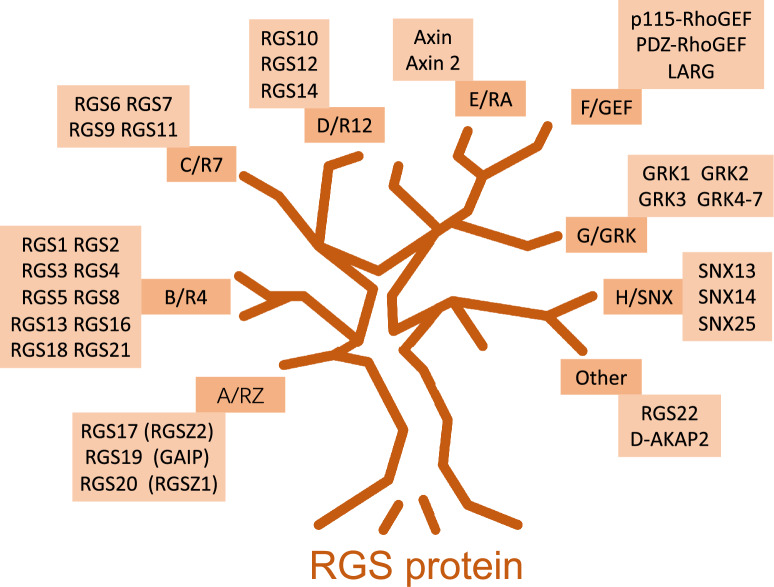
An abridged general view of the classification of RGS protein family. RGS proteins are divided into at least eight sub-classes based on sequence homology, with or without functional RGS homologous domains

To date, at least 37 different RGS proteins have been successfully discoveried and defined, with an extra 14 RGS proteins containing the domain that is nominated as the nonfunctional RGS homology domain [2], while the RGS proteins that posses a functional RGS domain can be divided into eight sub-families at a minimum [2]. As is depicted in Fig. 1, there are two kinds of systems for classifying RGS proteins, among which one system sorts sub-families via alphabet A to alphabet F, and the names of subfamily in the other classifying system are derived from the typical RGS member [3, 4]. For instance, the RZ family is nominated by the typical RGSZ protein [3, 5]. Based on the identity, structure and function of amino acid (AA) sequence, each family grouping is sub-sorted. For example, members belonging to the family named C/R7 possess a peculiar Disheveled, EGL-10, Pleckstrin (DEP) domain, which is called the R7H domain, whereas the A/RZ RGS proteins and B/R4 RGS proteins posses little more than a functional RGS domain. As a member in R4 family, RGS2 contains the simplest AA sequence structure [6]. RGS2-like proteins, including RGS1, RGS4, RGS5, RGS10, RGS13, RGS16, RGS18, and RGS-GAIP, belong to the small subfamily, which have little or almost no affinity for Gα-GDP complexes. In contrast, those proteins show high affinity in their binding to Gα-subunit complex particularly with AlF4 and GDP, contributing to a conformation of the Gα-subunit that features the interim condition causing hydrolysis of GTP. In rats and humans, the gene locus of *RGS2* is located in chromosome 1 and is consists of five exons. The *RGS2* locus, encoding a 212-residue protein, contains an RGS domain with120 AA that is sided by an N-terminal domain with 80-residue and a C-terminal tail of short length [7], which is similar to other RGS proteins in B/R4 class [6]. Of note, RGS2 protein owns the intrinsic GAP activity, which is vigorous and optional for Gq-class Gα-subunits in vitro, whereas other B/R4-family RGS proteins, in contrst to RGS2, possess intrinsic GAP activity for both Gi/o-class and Gq-class Gα-subunits [8, 9].

The classical RGS domain is essential and adequate for Gα-subunits binding. A previous report showed a crystal structure of the RGS domain in the RGS4 complex with Giα1 and GDP-AlF4, supporting the comprehension of how RGS proteins boost GTP hydrolysis [9]. The RGS domain is found to bind to the three switches area in Giα, and those binding-regions receive conformational changes upon their binding with guanine nucleotide and their hydrolysis. As has been proved, the RGS domain is comprised of nine α-helices that are further folded into two sub-domains of small size, and one small sub-domain shapes a structure with right-handed anti-parallel four-helix bundle. In addition, three parts with high conservation located in the RGS domain straightly link to the Giα switch spots, while mutations in these parts greatly interfere with the GAP viability of RGS protein. More recently, the results from structural and biochemical researches additionally reveal that RGS proteins spur Gα GTP hydrolysis through decreasing the energy of transitional state, leading to the destabilization of the enzyme substrate complex [9].

**Fig. 2:**
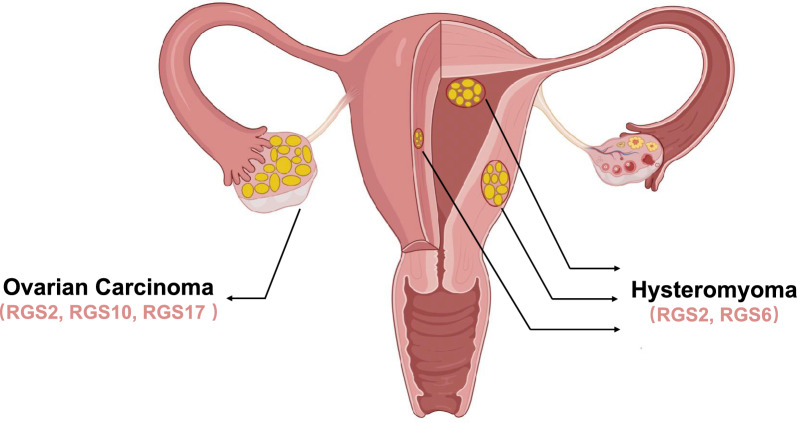
A diagram showing the female reproductive system. In this figure, bilateral ovarian tissue has obvious abnormal differentiation, the right ovarian tissue has obvious enlargement, and the ovarian tissue structure is heterogeneous, indicating the presence of ovarian cancer in this reproductive system. Abnormal spherical tissues of different sizes and degrees appeared in the uterine muscle wall, submucosa and serous membrane, respectively, indicating the presence of uterine fibroids in the uterus

Recently, emerging evidence has suggested that RGS proteins play a fundamental role not only in embryonic development but also in various diseases, including gestational disorders as well as gynecological diseases including gynecology malignancies [11–13], such as ovarian carcinoma and hysteromyoma (Fig. 2).

### Ovarian carcinoma

**Fig. 3:**
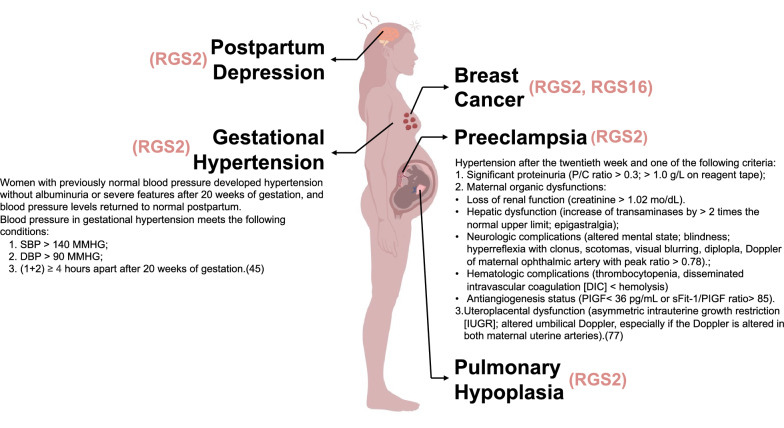
A diagram showing the gestational disorders during female pregnancy. Gestational hypertention, PE, postpartum depression are marked, and related diseases, including pulmonary hypoplasia and breast caner are also indicated. The woman in the picture shows signs of depression, which is a possible expression of postpartum depression. An abnormal lump structure in the breast tissue indicates breast cancer. Near the heart, a description of the woman’s abnormal blood pressure suggests that the woman has gestational hypertension. Abnormal proteinuria and other organic changes in the umbilical cord indicate that the woman is in a state of PE. The lung of the fetus in utero is marked with abnormal pulmonary dysplasia and signs of pulmonary fibrosis

As the mortality rate of patients has been over 60%, ovarian carcinoma is considered as one of the fatal gynecological cancers [14]. As is commonly believed, early detection contributes to elevating overall-survival, while only a quarter of ovarian cancers are discovered at stage I [15]. According to the previous researches, the development and existence of tumor cell resistance to chemotherapeutic drugs and the frequent diagnosis of ovarian carcinoma at an advanced stage are the major cause of high mortality rate [16]. Consistently, recent clinical data also indicated that low expression of RGS2 promotes poor prognosis in high-grade serous ovarian cancer (Fig. 3) [17].

GPCRs and epidermal growth factor receptors (EGFRs) are abundently expressed in ovarian carcinoma tissues [18]. G-proteins control GPCR-mediated cell survival signaling, which is opposite to the bioactivity of RGS proteins [19]. By straight association with the activated Gα subunit of G-proteins, RGS proteins can hasten the termination of GPCR signalings to raise hydrolysis of GTP into GDP, which returns G-proteins to a devitalized condition [20, 21]. Therefore, RGS proteins play an important role in cell survival and tumorigenesis. The aberrant expression of RGS2 is related to solid tumor development, and RGS2 expression is down-regulated in various cancers, such as ovarian cancer and prostate cancer [22–24]. Nevertheless, the molecular mechanisms underlying RGS2 expression inhibition are not clear yet. Therefore, inhibition of RGS2 may promote chemoresistance by EGFR or GPCR-mediated cell growth and cell survival signaling pathways.

Due to the accumulation of DNA methyltransferase 1 (DNMT1) and class I Histone deacetylases (HDACs) at the *RGS2* promoter region, the expression of *RGS2* gene is inhibited in ovarian cancer cells. Thus, these findings indicate that the epigenetic changes, including DNA methylation and histone modifications such as deacetylation and acetylation, may lead to the absence of *RGS2* expression in the ovarian cancer cells with chemoresistance. A recent research revealed the modulation of RGS2 expression by DNA methylation and histone deacetylation particularly in the chemoresistant ovarian cancer cells, and as a result, the expression of RGS2 is declined in the ovarian cancer cells resistant to drugs [24]. Thus, one conclusion can be made that drug exposure may give rise to the deficiency of *RGS2* expression during carcigogenesis in ovarian [25]. In addition, the acetylated histone H3 levels at the *RGS2* promoter are obviously reduced in chemoresistant ovarian cancer cells, compared with that in chemosensitive cells. However, the expression of histone H3 in ovarian cancer cells are similar to those in the chemosensitive cells, indicating that the acetylational absence at *RGS2* promoter leads to the deficiency of *RGS2* expression in ovarian cancer cells with chemoresistance [25]. Therefore, histone acetylation potentiates RGS2 expression, while HDACs antagonize it. On the other hand, class I HDACs can inhibit the expression of RGS2 in chemically blocked ovarian cancer cells [25]. These findings demonstrate that the increased HDACs (including HDAC1-3) binding in chemoresistant ovarian cancer cells corresponds to the reduction of histone acetylation at the *RGS2* promoter. Consistently, it has been shown that class I HDACs are abundantly distributed and widely expressed in ovarian cancer tissues, whereas abnormal HDAC expression is conserded to be closely associated with adverse response to chemotherapy [26, 27]. Therefore, HDAC inhibitors can be potentially employed to promote histone acetylation levels and to therefore elevate RGS2 expression levels, providing with the evidence that inhibition of histone deacetylase activity may be a new therapeutic target for epithelial ovarian cancers in clinic, particularly the platinum-resistant ovarian cancer [27].

Upregulation of methylation of tumor suppressor genes is often referred to as a potential biomarker for cancer progression [28, 29], and DNA methylation has also been found to be associated with ovarian cancer chemoresistance [23, 30–32]. DNMT1 is essential for maintaining the established DNA methylation patterns, whereas DNMT3a and DNMT3b normally promote the formation of de novo DNA methylation patterns [33]. Therefore, to maintain RGS2 repression during ovarian cancer progression, *RGS2* promoter might require the accumulation of DNMT1.

In addition to RGS2, other RGS proteins such as RGS5, RGS10 and RGS17 are also closely related to ovarian cancer [34]. Under certain hypoxic conditions, RGS5 expression is increased in ovarian cancer carcinoma-derived endothelial cells (ODMECs), and the proliferative capacity of ODMECs was significantly increased after targeted decrease of RGS5 expression. This suggests that RGS5 functions as a key mediator in the carcinogenesis of ovarian cancer [35]. Similarly, cellular deficiency in *RGS10* promotes chemoresistance in ovarian cancer [35]. RGS10 expression is regulated by DNMT1 and HDAC1, and dysregulation of RGS10 reduces chemoresistance in ovarian cancer cells [25, 34]. RGS17 can affect the occurrence and development of ovarian cancer through the PI3K/AKT cellular survival pathway [36]. Lysophosphatidic acid (LPA) can activate Gαi protein by binding to one of its receptors in an autocrine manner, which phosphorylates and subsequently activates protein kinase B (PKB, also known as AKT) and promotes cell survival consequently. The elevation in RGS17 expression resulted in a decrease in AKT activation followed by LPA treatment, thus illustrating the mechanism of growth arrest of ovarian cancer cells and the relevance of RGS17 expression in ovarian cancer cells.

In summary, since DNA methylation regulation is reversible, DNA demethylating agents may be an effective means of treating cancer in future and modulation of RGS2, RGS10 and RGS17 expression could be a promising approach to enhance the chemotherapeutic drug activity against drug-resistant cancer cells.

### Hysteromyoma

Hysteromyoma, known as the most common benign tumor in the female reproductive organs, are derived from the aberrant proliferation of uterine smooth muscle cells, where a small portion of fibrous connective tissue functions as a kind of support “material”. During pregnancy, due to the gestational function, uterus will undergo great changes in the first trimester. For instance, the volume and weight can increase by as much as 20 times compared to those before pregnancy [37]. Among these changes, the myometrium remains relatively static in order to ensure the normal development of the fetus and does not respond to uterine contractions. As a result, the myometrium is less sensitive to oxytocin through the relatively low levels of oxytocin receptor expression [38, 39]. Nevertheless, the levels of GPCR that regulate bradykinin, prostaglandins, angiotensin II, endothelin and hemolysin, and others, are not decreased obviously, suggesting that the myometrium does not perceive of endogenous concentrations of these drugs at the post-receptor level for most of the pregnancy. Part of the research suggests that the reason for non-perception is derived from the RGS2-induced reduction in GTPase activity. Due to the increased G-protein signaling of agonists and the decreased *RGS2* mRNA expression, uterine contraindications will take place at the end of pregnancy. Similarly, progesterone, an important progestin, it itself is important in upregulating *RGS2* mRNA expression. Therefore, *RGS2* mRNA expression can be prolonged by reducing the endogenous levels of progesterone, thereby treating uterine contraindications.

In addition to the above-mentioned regulation of GPCR signaling at the G protein expressing level, receptor insensitivity may be also expressed by means of specific kinases that phosphorylate residues in the carboxy-terminal domain of G protein-coupled receptors. Brenninkmeijer et al. reported that uterine tractions at the end of pregnancy in humans may be regulated by G protein-coupled receptor kinases 2 and 6 [40]. In this process, the uterotonic contractile agent will antagonize the effect of increase in the intracellular cAMP concentration [40]. During pregnancy, the expression of Gs in the myometrium is greatly increased and the potential to produce cAMP is enhanced [41], while the activity of Gs and its coupled adenylyl cyclase is decreased after delivery [42]. Despite that RGS2 indirectly inhibits GTPase activity, it has been reported that RGS2 directly suppresses adenylyl cyclase III activity in olfactory neurons [43]. However, since *RGS2* mRNA levels are minimal at term, this finding alone does not support the conclusion that RGS2 causes a decrease in adenylate cyclase activity at term. Given the association of discrete physiological events during pregnancy with changes in *RGS2* mRNA expression, it can be proposed that the myometrial RGS2 levels also participate in regulating uterine contractions [44]. However, some studies have indicated that the uterus is more responsive to stimulation by contractile agents [45]. In one study, this situation occurs when RGS2 expression is decreased. Therefore, it is likely that RGS2 exerts effects on regulation of myometrium.

Taken together, RGS2 plays a key role in regulating the myometrial response to progesterone. As uterine fibroids are a hormone-dependent tumor, the high hormone environment during pregnancy is one of the important factors causing uterine fibroids. The treatment of uterine fibroids through the regulation of RGS2 will undoubtedly have an important role in future research progress.

### Gestational hypertension

**Fig. 4:**
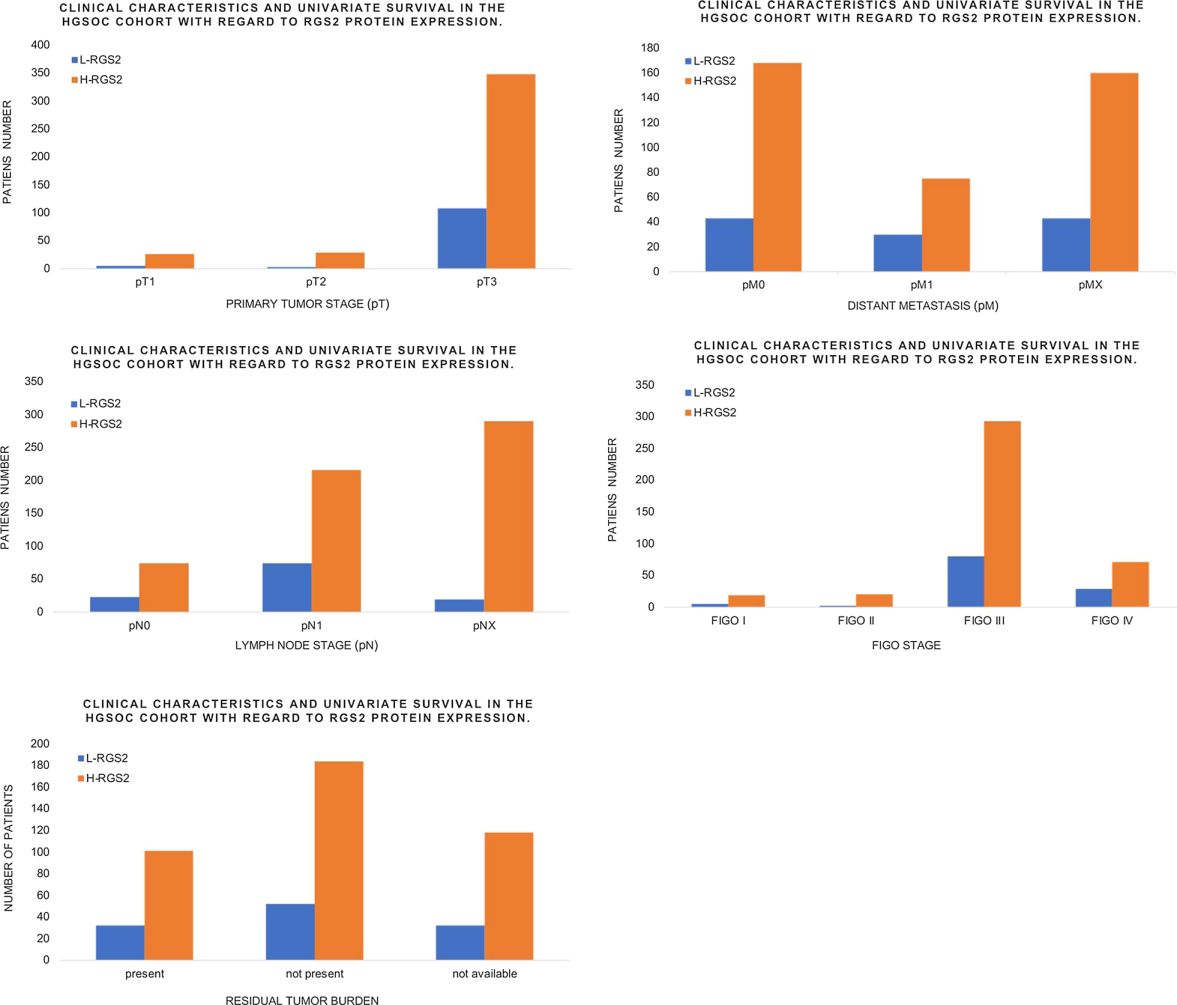
Clinical characteristics and univariate survival in the HGSOC cohort with regard to RGS2 protein expression

Gestational hypertension is diagnosed as two episodes of systolic blood pressure of 140 mm Hg or more, or diastolic blood pressure of 90 mm Hg or more, or both, at least 4 h apart after 20 weeks of pregnancy in women with normal blood pressure before pregnancy (Fig. 4) [46]. Women with hypertension is usually not accompanied by proteinuria or severe features, and can return to normal maternal blood pressure levels after delivery [46]. The main pathogenic manifestations of gestational hypertension are the systemic vasospasm and decreased multi-organ perfusion [47].

Lately, kinds of RGS B/R4 members have been demonstrated in vascular disorders of pregnancy. The cardiovascular system undergoes great changes during normal pregnancy. Cardiac output is remarkably increased, the renin-angiotensin system is activated, and angiotensin (ANG) is secreted when gestation is in week 24. In addition, arginine vasopressin (AVP) secretion is activated simultaneously in the same week. Both hormones take effect in the elevation of water absorption and retention, and as a result, the retained fluids can be raised to 45%, leading to a subsequent reduction in osmolality. During pregnancy, blood pressure can be also decreased to 5–10 mm Hg, and most of these changes appear early during the 6–8 week-range of gestation [48]. Pregnancy shows a general decrease in blood pressure that is considered to be affected by nitric oxide, progesterone or relaxin instead of the activation by vasoconstrictor hormones [48, 49]. Additionally, there are obviously reduced vasoconstriction responses to ANG during pregnancy [50].

RGS2 is widely distributed and expressed within human body. In addition to placenta, high expression levels of RGS2 are also detected in other tissues, such as brain, kidney and heart, and more recently, RGS2 expression is also found and tested in vascular smooth muscle cells (VSMCs) [51–53]. In addition, *Rgs2* is found to be expressed in certain regions in mouse brain, including the cortex, the striatum and the hippocampus as well. The expression levels of RGS2 are notably up-regulated by the activation of synaptic bioactivity and is involved in modulating the Gαq and/or Gαi-mediated activation of M2 muscarinic acetylcholine receptor (AChR) [54]. Both heterozygous (*Rgs2*^±^) knockout mice and homozygous (*Rgs2*^*−/−*^) knockout mice present a obvious phenotype of hypertension, making it hard to differentiate between the heterozygous (*Rgs2*^±^) and homozygous (*Rgs2*^*−/−*^) by simple observation [54]. Furthermore, lack of *Rgs2* (*Rgs2*^*−/−*^) leads to the enhancement in the inner thickness of renal arterial walls, which is a trait in humans carrying chronic hypertension [55]. The ratio of blood pressure decrease is significantly lowered in *Rgs2* knockout mice (*Rgs2*^*−/−*^) than that of wild-type (*Rgs2*^+*/*+^), with the administration of vasodilator agonists. In addition, treatment with vasoconstrictor in VSMCs where *Rgs2* is lost leads to the increase in peak calcium responses but the extended decrease in intracellular calcium levels post challenge [55]. *Rgs2* deficiency also mediates hypertension by increasing vascular tone, which is proved by the enhanced shrinkage of mesenteric obstacles and renal interlobar arteries, and by the elevated hypertension responses to ANG as well [55–57]. Despite that RGS2 mainly acts to restrain Gαq signaling in VSMCs, RGS2 is found to modulate Gαi/o signaling also in the vascular endothelium. Gαi/o signaling suppresses vasodilation, and absence of *Rgs2* leads to the enhancement of Gαi/o signaling and the abnormal vasodilation in exposure to acetylcholine [57]. Additionally, RGS2 is demonstrated to modulate Gαs signaling. Nevertheless, RGS2 fails to interact with Gαs itself. Recently, RGS2 has been shown to restrain the generation of cyclic adenosine monophosphate (cAMP), particularly in olfactory membranes of Gαs, and as a result, RGS2 adjusts adenylyl cyclase activity. Among the multiple adenylyl cyclases, RGS2 is identified to particularly modulate several adenylyl cyclases, including adenylyl cyclases III, adenylyl cyclases V, and adenylyl cyclases VI [58]. On the other hand, RGS2 is found to regulate the AVP-induced V2 receptor (Gαs)-mediated signaling. Within the kidneys, expression distribution of RGS2 is limited to the area of nephron where V2 receptors are expressed, and as the major signaling mediator of V2 receptors, the levels of cAMP are markedly up-regulated in kidneys from *Rgs2* knockout mice (*Rgs2*^*−/−*^). Accumulating evidence has additionally shown that AVP [59–61], endothelin-1 (ET-1) [62, 63], and ANG II [62, 64, 65] participate in the occurrence and progression of cardiovascular disorders during female gestation such as the hypertension syndrome. The main receptor subtypes that involved in cellular signaling transduction, such as V1A [47, 66–68], V2 [69], ETA/ETB [70–72] and AT1 receptors [73, 74], can respond to these vascular hormones, respectively, to activate G proteins and subsequently their corresponding second-messenger cascades in target cells and specific tissues. Regulators of G protein signaling or downstream mediators may share a generally common mechanism underlying cardiovascular disorders during gestation and thereby hopefully provide with potential targets for more promising therapy of gestational cardiovascular disorders in clinic.

### Preeclampsia (PE)

PE is a common complication in pregnancy, and there is substantial evidence that the reduced uteroplacental blood flow in this condition is due to a toxic combination of hypoxia, inflammation, an imbalance of angiogenic and antiangiogenic factors, and immune disturbances [75, 76]. In addition, women who suffer from PE also have an increased risk in cardiovascular and renal diseases [77, 78].

**Fig. 5:**
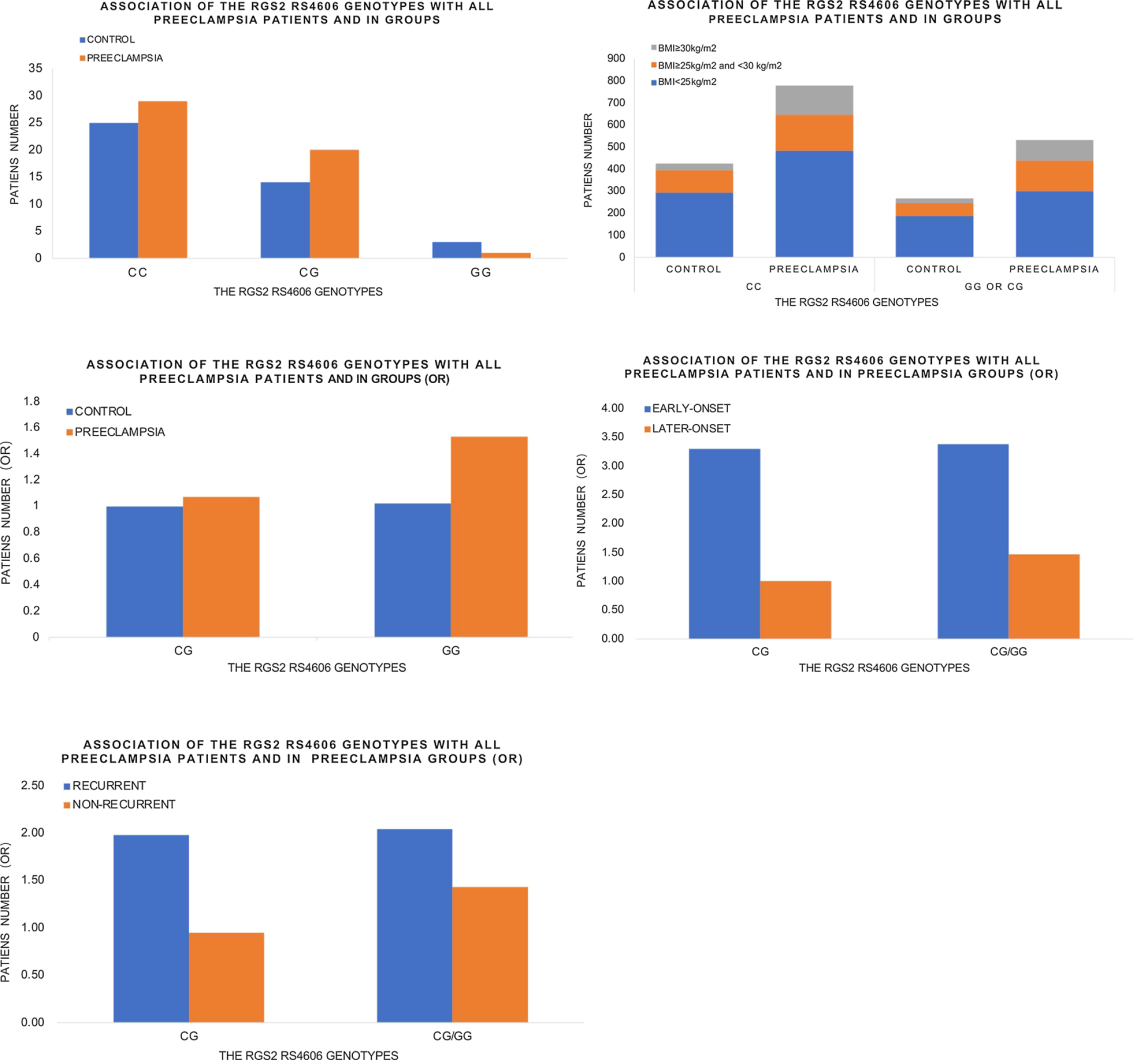
Association of the RGS2 expression with all preeclampsia patients and in groups

Recently, RGS2 has been found to be linked to PE and data from several clinical studies revealed that RGS2 expression was dysregulated in pregnant women with PE (Table 1 and Fig. 5) [79–81]. Researchers found that hypertension in selected human populations, a single nucleotide polymorphism (SNP, rs4606) located in the 3’-untranslated region (UTR) of *RGS2* gene is correlated with PE [77] and the same SNP (rs4606) is closely associated with an increased risk in PE development, particularly in a population of overweight females [82–84]. Moreover, women that carry the SNP (rs4606) are at increased odds of having PE and cardiovascular disease later in life [85]. Down-regulated levels of *RGS2* transcript expression has been found to be correlated with the SNP in the 3’-UTR of the *RGS2* gene [80]. Noticeably, as is discussed previously [82], *Rgs2* knockout mice (*Rgs2*^*−/−*^) and *Rgs2* heterozygous mice (*Rgs2*^±^) both reveal the hypertensive phenotypes, indicating *Rgs2* gene may exhibit haplo-insufficiency. Furthermore, both the homozygotes (*Rgs2*^*−/−*^) and heterozygotes (*Rgs2*^±^) exhibit alterations in the rs4606 C1114G SNP that are associated with the higher possibilities of PE development in human.

**Table 1:** Maternal and perinatal background characteristics of the study groups

Parameter	Control	Preeclampsia	P values
Age (years)	41.2	41.0	0.588
Body mass index, kg/m^2^	25.8	27.9	< 0.001
Pregnancy duration at delivery in weeks, mean	39.8	38.8	< 0.001
Highest systolic blood pressure (mmHg)	164.2	168.0	< 0.001
Highest diastolic blood pressure (mmHg)	102.2	105.0	< 0.001
Pregestational diabetes mellitus, %	1.0(20)	3.1(29)	< 0.001
Current smoker, %	38.4(741)	26.5(234)	< 0.001
Age (years)	20.0	30.0	0.020
Body mass index, kg/m^2^	23.6	23.0	0.006
Pregnancy duration at delivery in weeks, mean	40.6	37.9	< 0.001
Highest systolic blood pressure (mmHg)	126.0	165.0	< 0.001
Highest diastolic blood pressure (mmHg)	83.0	109.0	< 0.001
Pregestational diabetes mellitus, %	7.7(29)	11.8(116)	0.030
Current smoker, %	28.0(371)	58.0(948)	0.345
Age (years)	28.8	28.6	0.839
Body mass index, kg/m^2^	26.9	30.5	0.006
Pregnancy duration at delivery in weeks, mean	39.8	36.8	< 0.001

Pregnancy arouses physiological reshape of the cardiovascular system to help the organism keep usual blood pressure and normal organ perfusion, in case of facing the elevated maternal extracellular fluid volume [86, 87]. During pregnancy, notably increased levels of vasoactive hormones, particularly ANG II, are able to increase peripheral resistance, blood pressure, vasoconstriction, and sodium retention. Nevertheless, such effects usually are countered by up-regulating the generation of endothelium-derived relaxing factors, primarily nitric oxide (NO) [88]. Moreover, due to the effects caused by ANG II, the arterial vasculature during gestation turns refractory to vasoconstriction [89], despite the possible molecular mechanisms involved remain elusive yet. Having said that, flaws in such compensatory mechanisms, thus, may result in PE and hypertension during female pregnancy [90, 91].

RGS2 have protective effects on compensatory mechanisms that hold blood pressure steady during gestational period, which could be impeded in PE. By activating the AT1 receptors coupled with Gq, ANG II can trigger vasoconstriction that potentiates the activity or expression of RGS2 or other Gq adjusting proteins offering possibly adaptive mechanisms that usually hold blood pressure steady during pregnancy. The pathophysiology of PE may be derived from the impairment of such adaptive mechanisms. Recent studies have clarified the G protein signaling pathways that are related to blood pressure modulation in preeclamptic females and normotensive pregnant females, and have confirmed that, the *Rgs2* SNP (rs4606) located in the 3’-UTR is connected with progression and risk in PE [79]. Further evidence has shown that women carrying this *RGS2* SNP and suffering PE have the increased probability of developing hypertension after delivery [92]. More recently, work from our group elucidates that RGS2 regulates trophoblast cells epithelial-mesenchymal transition (EMT) in human placenta, and dysregulation of RGS2 is closely associated with hypoxia that contributes to PE [93, 94]. That being said, in order to discover how G protein signaling is precisely controlled and functionally remodeled during gestation, more work is in need to answer such questions as: 1) whether RGS2 is a significant component of this control mechanism? And: 2) whether RGS2 may provide with a promising therapeutic target for PE in clinic?

### Postpartum depression

Postpartum depression (PPD) is a frequent and serious psychological health disease that is associated with pregnant women’s pain and has lots of negative consequences for descendants. The half year after delivery is considered as a high-risk period for depression, with the prevalence rate from 13 to 19% [95]. Current research on antidepressant therapy and pathogenesis of depression is focused on the depression-like behavior phenotype [96], and some researchers have discovered that the mice with depression-like behavior have a high level of *Rgs2* expression [97]. In contrast, down-regulation of *Rgs2* relieved mouse cognitive impairment, and consistently, siRNA-mediated knockdown of *Rgs2* reinforced the 5-hydroxytryptamine (5-HT) levels in hippocampal CA1 neurons. Tryptophan is the source of 5-HT, which is synthesized both centrally and systemically [98], while 5-HT is recognized as one of the significant neurotransmitters in the pathogenesis of postpartum depression. Besides, it is reported that 5-HT receptors are underlying targets for treatment of cognitive impairment in senescence [99], and consistently, activity of cAMP pathway is elevated upon silencing of *RGS2* [99]. This mechanism can prevent cognitive impairment in mice with depressive behavior and enhance the regeneration of hippocampal neurons [99]. Consequently, silencing of RGS2 decreases oxidative stress injury and inflammation, but increases 5-HT concentration and cAMP pathway activity, thereby alleviating the cognitive impairment and neuronal damage in mice with depression-like behaviors. From this point of view, it can be suspected that RGS2 may play a role in the pathogenesis of PPD. However, the mechanisms are not fully clear yet, which is worth further investigation.

### Breast cancer

Although breast cancer (BC) is not belonging to diseases in gynaecology and obstetrics, it is one the most frequently diagnosed life-threatening cancer in women. We thereby herein also discuss the potential relationship between RGS and BC.

Now, it is well known that maternal BC can have significant effects on both the pregnant woman and the newborn due to the breastfeeding after newborn deliveries. BC is a terrible female disease with the high morbidity and mortality, which seriously affects the quality of women's life. A previous study reported that miR-183-5p could worsen BC progression through regulating RGS2. Moreover, RGS16, belonging to the same family, is indicated to be associated with BC, and *RGS16* is considered as a potential susceptibility gene in BC [100]. In a large number of BC, it has been shown that the allelic imbalance rate at the *RGS16* locus (1q25.3) is about 50%. Chromosomal breakpoints are predominantly represented by microdeletions in the *RGS16* promoter region, and the *RGS16* promoter is methylated in 10% of these tumors. In addition, almost two-thirds (∼67%) of the tumors with this mutation lead to the reduction in *RGS16* expression [101]. It is therefore very likely that RGS16 is closely related to the occurrence and development of BC during pregnancy and childbirth, with potentially corresponding values in the future.

### Pulmonary hypaoplasia

Gestational disorders during pregnancy in maternal body would give rise to neonatal healthy problems, such as pulmonary hypoplasia, and some previous reports have found that RGS2 is also involved in the growth and development of neonatal lung.

To ensure the fetus to develop and grow well outside the body during childbirth, the neonatal lungs must provide sufficient volume and surface area for gas exchange. During all stages of lung growth and development, the dynamic activity of fetal respiratory movement and the accumulation of pulmonary fluid are the top priorities. RGS2 suppresses the signals such as duration and amplitude, which are modulated by G_q_-coupled GPCRs [102–104]. Intriguingly, some G_q_-coupled GPCRs and their corresponding ligands are significantly actuated of pulmonary fibrosis, such as lysophosphatidic acid receptor 1, protease-activated receptor-1 (PAR1), and endothelin receptors [105–108]. Repression of these signals by RGS2 that is up-regulated by pirfenidone (PFD) thus provides with a potentially mechanistic argument for the useful effects of both PFD and RGS2 in terms of decreasing fibrotic reacts of lung fibroblasts. As a matter of fact, when thrombin is contacted with human fetal lung fibroblast 1 (HFL1) cells, a protease is increased in bronchial alveolar lavage fluid of idiopathic pulmonary fibrosis (IPF) patients [109], while direct up-regulation of RGS2 expression to the levels comparable to those increased by PFD treatment can cause a series of anti-fibrotic responses in human lung HFL1 cells. Therefore, discontinued PFD treatment or RGS2 inhibition can activate the thrombin-induced differentiation and proliferation of HFL1 cells, indicating that HFL1 cells are a key component of IPF [110]. In contrast, PFD treatment or overexpression of RGS2 restrains the thrombin-induced collagen production, connective tissue growth factor (CTGF) expression, and gel shrinkage as well. Moreover, overexpression of RGS2 significantly reduces thrombin-induced intracellular Ca^2+^ transportation [110], whereas the up-regulated intracellular Ca^2+^ promotes the proliferation and differentiation of fibroblasts, induces fibroblast-to-myofibroblasts conversion [111], leads to apoptosis of type II lung cells [112], and ultimately gives rise to the formation of pulmonary fibrosis. To sum up, despite that the relevant mechanism is not clear and needs further research and demonstration, it can be naturally speculated that a premature infant may be short of RGS2 expression in pregnant women, leading to the occurrence of pulmonary hypoplasia during pregnancy.

### Other diseases associated with women’s health

As an important signaling protein, RGS2 is closely related to the occurrence and development of obstetrics and gynecology diseases. In addition to the research reports on diseases mentioned above (Table 2), RGS2 is also found to play an important role in other systemic diseases, such as prostate cancer and cardiac hypertrophy [113], where RGS2 expression levels are dysregulated. Moreover, up-regulated expression of RGS2 causes reduced insulin signaling in human endothelial cell lines, which is associated with poorly controlled diabetes [114], suggesting RGS2 expression would be also linked to gestational diabetes mellitus, known as one of the most common medical complications of pregnancy. Recent researches have also indicated that gastric cancer and colorectal cancer are related to *RGS2*. A large amount of RGS2 is deposited in the matrix of gastric cancer [115] and the down-regulation of RGS2 is involved in the metastasis of colorectal cancer [116], indicating the possibility of RGS2 as a therapeutic target in cancer. These abdominal malignant tumors have the potential to metastasize and infiltrate the female reproductive system, thus affecting women’s health consequently. Lately, some studies demonstrate that the dynamic regulation of RGS2 is a potential mechanism for thyroid hormone regulation, which is the main cause of high incidence of thyroid sarcoidosis in women [117, 118], reinforcing the notion that RGS2 expression is strictly and precisely controlled depending on the specific context within human body. Therefore, continuously in-depth work on RGS2 protein is in need to fully figure out the function and molecular mechanisms underlying RGS2 regulation in different diseases by studies as well as experiments in vitro.

**Table 2:** A table summarizing the function of different RGS proteins in female disorders/diseases discussed in this paper

Disease	RGS protein	Mechanisms	References
Ovarian carcinoma	RGS2 RGS5 RGS10 RGS17	Accumulation of DNMT1 and class I HDACs Histone modifications and DNA methylation To regulate PI3K/AKT survival pathway	[24–26, 30, 34]
Hysteromyom	RGS2 RGS6	To reduce the endogenous levels of progesterone	[39, 42, 44]
Gestational hypertension	RGS2	To restrain Gαq signaling in vascular smooth muscle To regulate Gαi/o signaling in the vascular endothelium To alter Gαs signaling	[55, 72]
Preeclampsia	RGS2	SNP (rs4606) is correlated with an increased risk in PE development To activate Gq-coupled AT1 receptors, ANG II can trigger vasoconstriction To regulate trophoblast cells epithelial-mesenchymal transition Regulated by hypoxia that contributes to PE	[78, 89, 90]
Postpartum Depression	RGS2	Knockdown of Rgs2 reinforces the 5-HT levels in hippocampal CA1 neurons Silencing of Rgs2 accelerates the cAMP pathway activation	[96, 98]
Breast cancer	RGS2 RGS16	miR-183-5p promotes BC progression through RGS2 DNA methylation by microdeletions in the *RGS16* promoter region (1q25.3)	[12, 100]
Pulmonary Hypoplasia	RGS2	To repress signals such as duration and amplitude To reduce thrombin-induced intracellular Ca2 + signaling	[105, 106, 109]

## Conclusion and perspective

Owing to the efforts in past decades, we have obtained a better understanding on RGS2 and its role in diseases in obstetrics and gynecology. Given that the alterations in RGS2 expression levels and/or functions consequently result in various implications involved in certain diseases, and that some small chemical molecules such as the compound (1-(5-chloro-2-hydroxyphenyl)-3-(4-(trifluoromethyl)phenyl)-1 H-1,2,4-triazol-5(4 H)-one) tested to be a Gαq-RGS2 signaling inhibitor by animal experiments [119], RGS2 is likely to be a promising drug target for human diseases in future. Thus, identifying viable small molecule drugs that target RGS2 would hopefully contribute to the intervention and treatment of related diseases.

## Data Availability

All data generated or analyzed during this study are included in this published article.

## Abbreviations

5-HT: 5-Hydroxytryptamine
AA: Amino acid
AChR: Acetylcholine receptor
ANG: Angiotensin
AVP: Arginine vasopressin
BC: Breast cancer
Camp: Cyclic adenosine monophosphate
CTGF: Connective tissue growth factor
DBP: Diastolic blood pressure
DEP: Disheveled, EGL-10, Pleckstrin
DNA: Deoxyribonucleic acid
EGFRs: Epidermal growth factor receptors
EMT: Epithelial-mesenchymal transition
ET-1: Endothelin-1
GAP: GTPase activating protein
GDP: Guanosine diphosphate
GPCR: G protein-coupled receptor signaling
GTP: Guanosine triphosphate
GTPase: Guanosine triphosphatase
HFL1: Human fetal lung fibroblast 1
IUGR: Intrauterine growth restriction
IPF: Idiopathic pulmonary fibrosis
LPA: Lysophosphatidic acid
mRNA: Messenger RNA
NO: Nitric oxide
ODMECs: Ovarian cancer carcinoma derived endothelial cells
PAR1: Protease-activated receptor-1
PE: Preeclampsia
PFD: Pirfenidone
PIH: Pregnancy induced hypertension syndrome
PlGF: Placental growth factor
PKB: Protein kinase B
PPD: Postpartum depression
P/C: Urinary protein/creatatine
RGS: Regulator of G-protein signaling
RGS2: Regulator of G-protein signaling 2
RNA: Ribonucleic Acid
SBP: Systolic blood pressure
sFLT-1: Soluble fms-like tyrosine kinase-1
SNP: Single nucleotide polymorphism
